# Probiotics: multifunctional microorganisms for human health and biotechnological applications

**DOI:** 10.3389/fmicb.2026.1847515

**Published:** 2026-05-29

**Authors:** Mushtaq Ali, Alok Srivastava, Pankaj Kumar Arora

**Affiliations:** 1 Department of Microbiology, MJP Rohilkhand University, Bareilly, India; 2 Department of Plant Science, MJP Rohilkhand University, Bareilly, India

**Keywords:** antimicrobial activity, clinical applications, gut microbiota, immune modulation, probiotics

## Abstract

Probiotics are live microorganisms that, when ingested in sufficient amounts, can have a beneficial impact on health. As crucial agents in maintaining gut homeostasis, enhancing immunity, and preventing of numerous diseases, they are fundamentally important. Probiotic function is based on pathogen inhibition, the release of antimicrobial substances, immune modulation, and the enhancement of the intestinal barrier integrity. Technological advances in the area, including molecular identification, microencapsulation methods, and metagenomics, have also been discussed. In addition, research methodologies for several subclasses of probiotics including *Lactobacillus* and *Bifidobacterium* continually being investigated. The role of probiotics in health of human, along with existing challenges related to probiotic viability and strain specificity, has also been discussed. This review highlights the growing understanding of probiotics and underscores their potential for optimizing human health and therapeutic applications.

## 1 Introduction

Antimicrobial resistance (AMR) is arguably one of the most serious global health threats, jeopardizing the efficacy of antibiotics. It was directly responsible for 1.27 million fatalities and contributed to almost 4.95 million deaths worldwide in 2019. By 2030, unrestrained AMR is anticipated to decrease the global Gross Domestic Product (GDP) by $1–3.4 trillion each year, with low- and middle-income countries bearing the brunt of the burden (Aslam and Aljasir, 2025; Hussain et al., 2025a). The overconsumption and misuse of antimicrobials in human, veterinary, and agricultural activities have facilitated the more rapid spread of non-susceptible pathogens. The current antibiotic pipeline is insufficient because there are few new compounds in development and no new classes of antibiotics have been discovered in recent decades. Of further concern are the resistance trends in opposition to final resort medications such as carbapenems, which complicate the management of common infections like pneumonia and urinary tract infections. In this situation, probiotics have received increasing attention because they could help minimize or replace antibiotics. Probiotics are live bacteria that, when administered in adequate amounts, confer some health benefits to the recipient, according to World Health Organization and Food and Agriculture Organization. Through altering gut microbiota, boosting immunity, and direct competition against pathogens in terms of sources of nutrition and adhesion points, probiotics provide a way to reduce infection cases and use of antibiotics (Aslam and Aljasir, 2025; Oliveira et al., 2024). Probiotics follow three essential criteria: the microorganisms must be viable, administered in adequate amounts, and confer scientifically demonstrated health benefits to the host (Thilagavathi, 2020). Probiotics are evaluated to ensure safety, including precise taxonomic description, comprehensive safety examination, and assessment of at least one positive human trial, before being permitted in foods, dietary supplements, and therapeutic products (Helmy et al., 2023; Reid et al., 2019). Only those strains that meet all of the criteria may be used (Binda et al., 2020). This analysis aims to investigate the mechanisms by which probiotics contribute to human health and biotechnology. Specifically, it describes their mechanisms of action, clinical confirmation of their benefits, desired applications, as well as cutting-edge methods such as CRISPR-dependent engineering and encapsulation. Finally, its strain-dependent constraints, hurdles to reproducibility, and concerns about safe use are discussed, as well as an outlook for the future of the field and medically relevant probiotics.

## 2 Common probiotic bacterial genera

*Lactobacillus*, *Bifidobacterium*, *Saccharomyces*, and *Streptococcus* are the most studied probiotic genera. *Lactobacillus* has been utilized in studies due to the multiple beneficial influences they have on the gut. These include the organism’s ability to assist in protecting against infections from pathogenic gastrointestinal organisms (Ding et al., 2024; Rustamovna, 2025). The *Bifidobacterium* also has a important role in digestion and immune modulation. Among yeasts, the *Saccharomyces* in particular, *Saccharomyces boulardii* is famous as an antifungal and its contribution to gut health (Nie et al., 2025). *Streptococcus* is another commonly employed probiotic, linked to gut and immune health and discovered in fermented milk products like yogurt (López-Palestino et al., 2025). The effects of probiotic strains vary and include immunomodulation, maintenance of microbial homeostasis, and pathogen inhibition (Sudheer et al., 2025). Probiotics are naturally linked to dairy products due to the organisms’ improved survival and stability in such foods (Sahatsanon et al., 2024). They are, however, also accessible in formulated forms like pills, powders, and functional nutrition, requiring verification of microbial viability throughout the product shelf life (Fancher et al., 2020; Dapkevicius et al., 2021). A listing of many of the common probiotic organisms is available in Table 1.

**Table 1:** Most commonly used probiotic microorganisms.

Probiotic genera	Probiotic strains	References
*Lactobacillus*	*Lactobacillus acidophilus* *Lactobacillus amylovorus* *Lactobacillus bulgaricus* *Lactobacillus crispatus* *Lacticaseibacillus casei* *Lactobacillus gasseri* *Lactobacillus helveticus* *Lactobacillus johnsonii* *Lactiplantibacillus pentosus* *Limosilactobacillus reuteri* *Lacticaseibacillus paracasei* *Lactiplantibacillus plantarum* *Lacticaseibacillus rhamnosus*	Islam (2016), Ooi et al. (2015), Dixit et al. (2016), Westermann et al. (2016)
*Bifidobacterium*	*Bifidobacterium animalis* *Bifidobacterium breve* *Bifidobacterium infantis* *Bifidobacterium bifidum* *Bifidobacterium lactis* *Bifidobacterium catenulatum* *Bifidobacterium longum* *Bifidobacterium adolescentis*	Bartosch et al. (2005), Macfarlane et al. (2008), Aachary and Prapulla (2011)
*Enterococcus*	*Enterococcus faecium*	Ali et al. (2022)
*Streptococcus*	*Streptococcus thermophilus*	Arora et al. (2013)
*Lactococcus*	*Lactococcus lactis* *Lactobacillus curvatus*	El Jakee et al. (2016)
*Bacillus*	*Bacillus clausii* *Bacillus coagulans* *Bacillus subtilis* *Bacillus laterosporus*	European Food Safety Authority (EFSA) (2017), Nguyen et al. (2016)
*Pediococcus*	*Pediococcus acidilactici* *Pediococcus pentosaceus*	Sornplang and Piyadeatsoontorn (2016)
*Propionibacterium*	*Propionibacterium jensenii* *Propionibacterium freudenreichii*	Dixit et al. (2016)
*Streptococcus*	*Streptococcus sanguis* *Streptococcus oralis* *Streptococcus mitis* *Streptococcus thermophilus* *Streptococcus salivarius*	Arora et al. (2013)
*Bacteroides*	*Bacteroides uniformis*	Kobyliak et al. (2016)
*Peptostreptococcus*	*Peptostreptococcus productus*	Nguyen et al. (2016)
*Escherichia*	*Escherichia coli* Nissle 1917	European Food Safety Authority (EFSA) (2017)
*Faecalibacterium*	*Faecalibacterium prausnitzii*	Giorgetti et al. (2015)
*Akkermansia*	*Akkermansia muciniphila*	Kobyliak et al. (2016)
*Saccharomyces*	*Saccharomyces cerevisiae, Saccharomyces boulardii*	Chen et al. (2013)

### 2.1 Next,-generation probiotics (NGPs)

Beyond traditional strains, emerging species known as next-generation probiotics (NGPs) are gaining attention. Notable examples include *Akkermansia muciniphila* and *Faecalibacterium prausnitzii,* both of which play key roles in metabolic regulation, immune modulation, and inflammation control (Fijan, 2023; Al-Jebory et al., 2023). Unlike conventional probiotics, these organisms require specialized cultivation and are not yet widely available in commercial products, but they represent promising candidates for future therapeutic development.

### 2.2 Prebiotics and synbiotics

Probiotic efficacy can be enhanced when combined with prebiotics, which are non-digestible fibers that selectively stimulate beneficial bacteria in the gut. Common prebiotics include fructo-oligosaccharides (FOS), galacto-oligosaccharides (GOS), inulin, lactulose, and resistant starches (Sabater-Molina et al., 2009; Slavin, 2013; Patel and Goyal, 2012; De Souza Oliveira et al., 2011; Zhou et al., 2014; Torres et al., 2010). The combination of probiotics and prebiotics is termed a synbiotic, designed to improve colonization and functionality of beneficial microbes. Examples include formulations of *Bifidobacterium* strains with inulin or *Lactobacillu*s with FOS. A detailed list of common prebiotics and their sources is provided in Table 2.

**Table 2:** Common prebiotics used in synbiotic formulations.

Prebiotics	Sources	References
Fructo-oligo-saccharides	Fructo-oligosaccharides Onion, Leek, Asparagus, Chicory, Jerusalem artichoke, Garlic, Wheat, Oat	Sabater-Molina et al. (2009)
Inulin	Agave, banana, burdock camas, chicory, coneflower, cestus, elecampane, globe artichoke, dandelion, Jerusalem artichoke, jicama, wild yam, mug wort root, garlic, onion	Slavin (2013)
Iso-malto-oligosaccharides	Miso, soy, sauce, sake, honey	Patel and Goyal (2012)
Lactulose	Skim milk	De Souza Oliveira et al. (2011)
Lacto-sucrose	Milk sugar	Zhou et al. (2014)
Galacto-oligo-saccharides	Lentil, human milk, chickpea/hummus, green pea, lima bean, kidney bean	Torres et al. (2010), Vulevic et al. (2013)
Soybean oligosaccharides	Soybean	Rycroft et al. (2001)
Xylo-oligosaccharides	Bamboo shoot, milk, honey	Aachary and Prapulla (2011)
Fructo-oligosaccharides	Onion, chicory, garlic, asparagus, banana, artichoke	Moro et al. (2006)
Arabinoxylan	Bran of grasses	Le Barz et al., 2015
Arabinoxylan oligosaccharides	Cereals	Grootaert et al. (2009)
Resistant starch-1,2,3,4	Beans, legumes, starchy fruits and vegetables,	Fuentes-Zaragoza et al. (2011)

## 3 Mechanisms of action of probiotics

**Figure 1:**
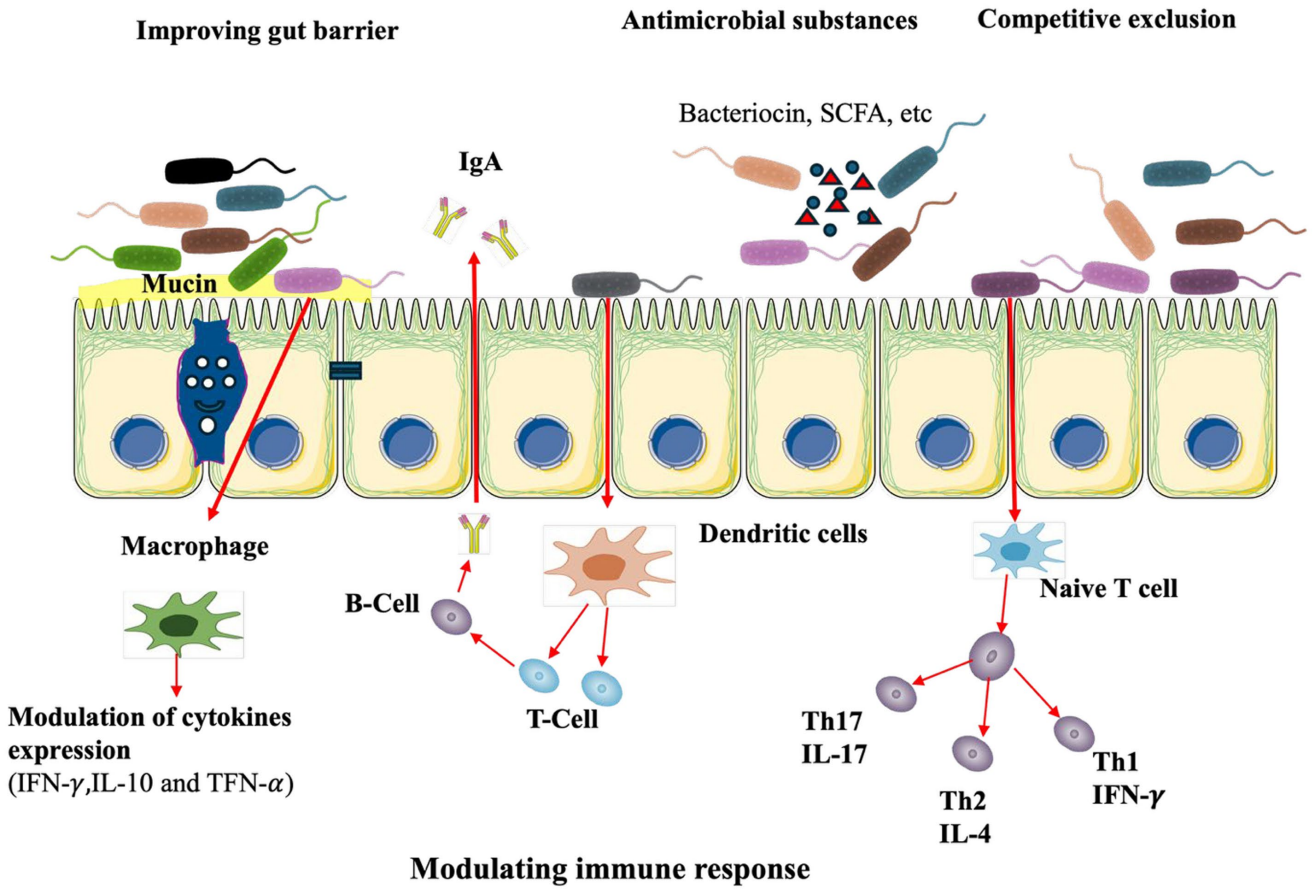
The figure shows the major mechanisms through which probiotics maintain gut health. Probiotics improve the gut barrier function by inducing mucin secretion and epithelial integrity. Probiotics produce antimicrobial compounds that suppress pathogenic microbes and exclude them from adhesion sites as well as nutrients (competitive exclusion). Probiotics also modulate the host’s immune response through interactions with immune cells and cytokine regulation, hence enhancing immune homeostasis and intestinal homeostasis.

Probiotics exert their beneficial effects through multiple mechanisms that maintain gut homeostasis, enhance host immunity, and suppress pathogenic microorganisms. The main mechanisms include competitive exclusion of pathogens, production of antimicrobial compounds, modulation of the gut environment, disruption of biofilms, interference with quorum sensing, and immunomodulation (Figure 1).

### 3.1 Competitive exclusion of pathogens

Probiotics exert their protective effects by competing with pathogenic microorganisms for adhesion sites and nutrients within the intestinal mucosa, thereby preventing the colonization of harmful bacteria (Simón et al., 2021; Fathima et al., 2022). For instance, *Lacticaseibacillus rhamnosus* GG has been shown to reduce *Salmonella typhimurium* colonization in poultry (Zhang et al., 2024), while *Lactobacillus acidophilus* helps decrease *Clostridium difficile* infections by restoring the balance of gut microbiota (Wu et al., 2022). Additionally, probiotics secrete bacteriocins, lactic acid, and hydrogen peroxide, which suppress pathogen survival (Monika et al., 2021; Darbandi et al., 2022; Soltani et al., 2021; Todorov et al., 2022). They are also important for reinforcing the intestinal barrier by modulating tight junctions and promoting mucin production, thus avoiding pathogen transfer. New studies have shed light on their importance in balancing epithelial cells and inflammation linked to barrier malfunctioning (Zhang T. et al., 2022; Zheng et al., 2023; Abdulqadir et al., 2026).

### 3.2 Production of antimicrobial substances

Probiotics generate diverse antimicrobial compounds that inhibit pathogenic growth.

#### 3.2.1 Bacteriocins

These bacteriocins are peptides synthesized by the ribosome, through destabilization of target cell membrane and interference with the vital processes occurring in the cell (Aghemwenhio and Omonigho, 2024). For instance, nisin produced by *Lactococcus lactis* has a strong inhibitory effect on *Listeria monocytogenes*, thereby widely applied as a natural food preservative (Johnson et al., 2018; Anjana and Tiwari, 2022). Similarly, the inhibitory activity of other bacteriocins such as plantaricin, pediocin, and subtilin on a range of pathogenic bacteria was reported. E.g., *Staphylococcus aureus, Escherichia coli*, and *Pseudomonas aeruginosa* personality (Gaspar et al., 2018; Lauková et al., 2018; Lei et al., 2022; Ghosh et al., 2019; De Giani et al., 2019; Wei et al., 2021). In Table 3, specific examples of bacteriocins and their target microorganisms are provided.

**Table 3:** Antimicrobial activity of some probiotic bacteriocins.

Bacteriocins	Probiotic	Target microorganisms	References
Bacteriocin	*Lactobacillus acidophilus* KS400	*Gardnerella vaginalis, Streptococcus agalactiae, Pseudomonas aeruginosa*	Gaspar et al. (2018)
Enterocin M	*Enterococcus faecium* AL41	*Campylobacter* spp. *Clostridium* spp.	Lauková et al. (2018)
Nisin-like bacteriocin	*Lactococcus lactis* C15	*Escherichia coli*	Lei et al. (2022)
Pediocin	*Pediococcus pentosaceus* GS4 (MTCC 12683)	*Staphylococcus aureus* (ATCC 25923), *Escherichia coli* (ATCC 25922), *Pseudomonas aeruginosa* (ATCC 25619), and *Listeria monocytogenes* (ATCC 15313)	Ghosh et al. (2019)
Plantaricin P1053	*Lactiplantibacillus plantarum* PBS067	*Staphylococcus aureus* and *Escherichia coli*	De Giani et al. (2019)
Subtilin-like bacteriocin Subtilin JS-4	*Bacillus subtilis* JS-4	*Listeria monocytogenes*	Wei et al. (2021)

#### 3.2.2 Organic acids

Lactic and acetic acids reduce intestinal pH, creating an unfavourable environment for pathogens while supporting beneficial microbes (Czech et al., 2022; Yadav et al., 2024; Cai et al., 2024).

#### 3.2.3 Hydrogen peroxide (H₂O₂)

Certain *Lactobacillus* species produce H₂O₂, which damages pathogen DNA and proteins, inhibiting growth of organisms such as *Staphylococcus aureus* and *Escherichia coli* (Nguyen, 2025; Kaewchomphunuch et al., 2022; Sharma and Lee, 2025).

### 3.3 Modulation of gut pH and nutrient competition

The reduction of carbohydrate content: By fermenting carbohydrates, probiotics lower intestinal pH below ~5.5. This provides a more favourable environment for butyrate-producing bacteria to grow while simultaneously disinfecting pathogens concern (Dasriya et al., 2024; Ayed et al., 2024; Connolly et al., 2024; Yamamura et al., 2023; Resta, 2009). Probiotic bacteria metabolize what is left of nutrient available, thereby denying pathogens nutrient for growth (Hosseini Nezhad et al., 2015; Mohan, 2015; Mathipa and Thanstsha, 2017). Acetate, propionate, and butyrate are among the short-chain fatty acids (SCFAs) that are created by fermentation of dietary fiber in the large intestine. Short-chain fatty acids have crucial functions in the maintenance of intestinal health since they regulate immunity, energy production, and provide assistance to the cells of the intestinal lining. Short-chain fatty acids are most concentrated in the lower gastrointestinal tract (Li et al., 2023a). Short-chain fatty acids (SCFAs) are produced by the colonic microbiota as a result of probiotic-mediated fermentation. SCFAs contribute to colorectal cancer prevention by serving as an energy source for colonocytes, strengthening gut barrier integrity, improving glucose metabolism, and reducing inflammation (Markowiak-Kopeć and Śliżewska, 2020; Barbara et al., 2021).

### 3.4 Biofilm disruption and inhibition

Pathogenic biofilms possess high resistance to antibiotics and immune system clearance; however, research studies have confirmed that probiotics can disrupt or prevent biofilm formation by numerous mechanisms (Amato et al., 2022; Flemming and Wingender, 2010; Elshobary et al., 2025; Tomé et al., 2023). For example, it is known that some *Lactobacillus* species can interfere with gene expression by downregulating biofilm-relevant genes in *Streptococcus mutans* and *Candida albicans* (Barzegari et al., 2020; Mishra et al., 2020). Furthermore, *Lacticaseibacillus rhamnosus* is capable of generating major extracellular biosurfactants that can destabilize or devastate biofilm structural integrity (Al-Shamiri et al., 2023; Ragupathi et al., 2024; Naseef Pathoor et al., 2024). Biofilms can also be inhibited through enzymatic depolymerization of exopolysaccharides secreted by *Lactobacillus acidophilus*, thus impeding attachment bacterial biofilms of pathogen *Escherichia coli* O157: H7 (Lin et al., 2021; Ma et al., 2022; Khani et al., 2024). These probiotic processes are practical for reducing certain healthcare-associated infections and biofilm development in various sectors, notably the food industry (Dawan et al., 2025; Deng et al., 2020).

### 3.5 Quorum sensing (QS) interference

**Figure 2:**
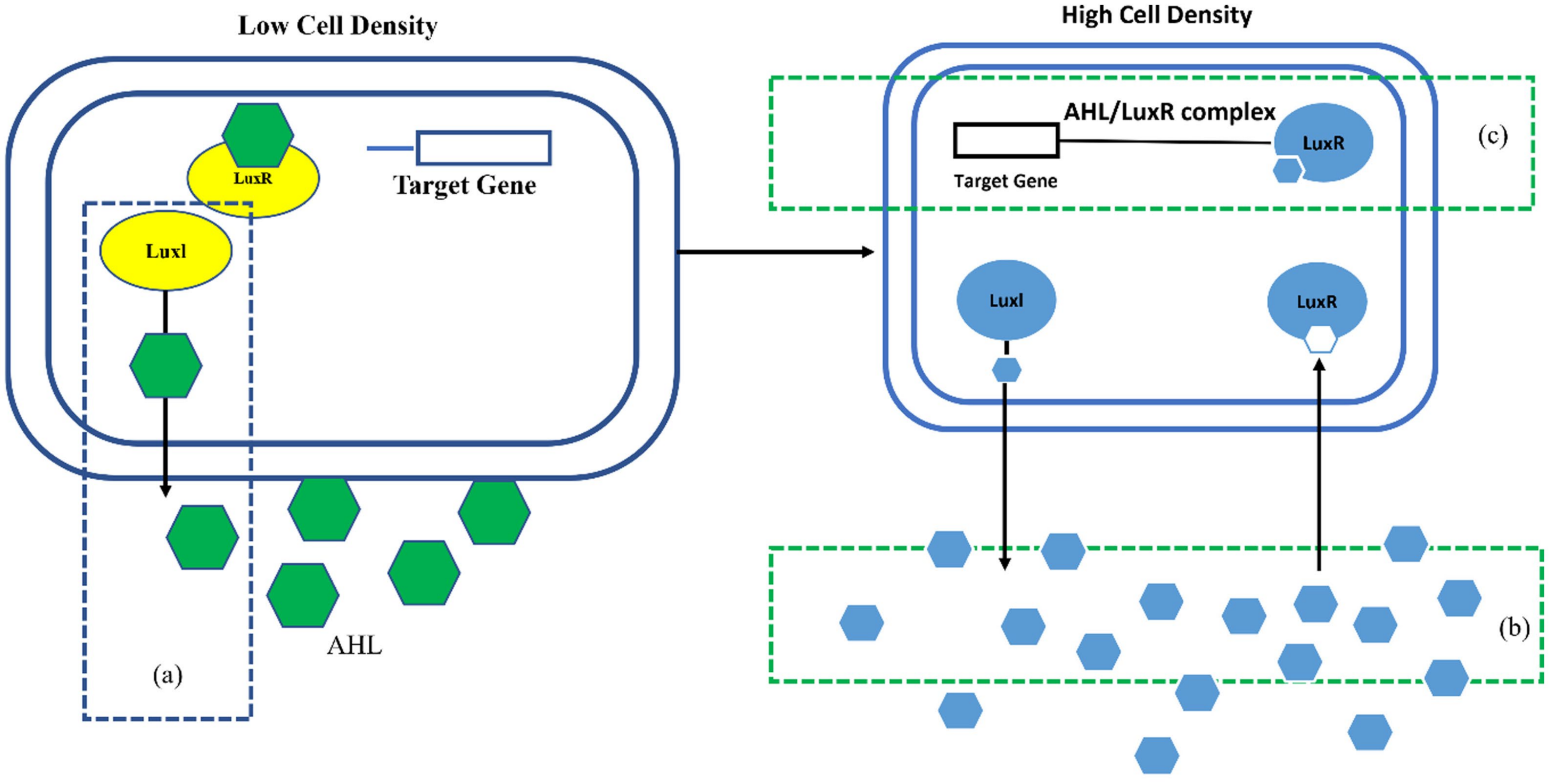
Quorum sensing in Gram-negative bacteria can be inhibited by: **(a)** Inhibiting the synthesis of acyl-homoserine lactones (AHLs); **(b)** enzymatic degradation of autoinducers (AIs); and **(c)** preventing the interaction of signalling molecules with their receptors.

In essence, QS constitutes a core regulatory mechanism that affects bacterial virulence, antibiotic resistance, and biofilm formation. Probiotics can interfere with QS via quorum quenching (QQ), which diminishes pathogenicity and enhances microbial equilibrium (Rattray et al., 2022; Raju et al., 2022; Li X. et al., 2021). Mechanisms of QQ include enzymatic destruction; namely, lactonases and acylases that break down acyl-homoserine lactones (AHLs) and autoinducing peptides (AIPs) (Markowska et al., 2024; Salman et al., 2023; Dicks, 2022). probiotics may also obstruct signal receptors, thereby forestalling AIs from attaching and, as a result, thereby preventing toxin production, as shown in Figure 2 (Su and ding, 2023; De Brito et al., 2022). In other situations probiotics act via the generation of inhibitory metabolites; organic acids and bacteriocins, which disrupt QS signal transport (Salman et al., 2023; Monika et al., 2021). Moreover, some probiotics competitively scavenge AIs, reducing signal accumulation and communication among pathogenic bacteria (Uhlig and Hyland, 2022; Juszczuk-Kubiak, 2024). Clinically, these quorum-quenching activities contribute to the attenuation of multidrug-resistant pathogens like *Pseudomonas aeruginosa* and enhance food safety by inhibiting spoilage organisms (Curcic et al., 2024; Davares et al., 2022; Zapaśnik et al., 2022; Vladkova et al., 2024; Hussain et al., 2025b). (Refer to Table 4 for examples of probiotic strains exhibiting quorum quenching activity.)

**Table 4:** Probiotic strains with QQ activity and the mechanism involved.

Genus	Species	Bacteria inhibited/fungus	QSI mechanism	References
	*Bacillus subtilis*	*Listeria monocytogenes, Escherichia coli, Gardnerella vaginalis*	Blocks AI-2, preventing biofilm growth	Kachhadia et al. (2022)
	*Bacillus cereus* RC1	*Lelliottia amnigena, Pseudomonas aeruginosa* MTCC2297	It reduces the creation of pyocyanin in *Pseudomonas aeruginosa* and alters the disease-causing ability of *Lelliottia amnigena*	Devi et al. (2018)
*Bacillus*	*Bacillus subtilis* R-18	*Serratia marcescens*	This bacterial extract prevents biofilm development and suppresses the output of protease, lipase, and hemolysin.	Boopathi et al. (2022)
	*Bacillus subtilis* BR4	*Pseudomonas aeruginosa*	Suppresses biofilm development.	Nithya et al. (2010)
	*Bacillus pumilus*	*Pseudomonas. aeruginosa* PAO1 (las, rhl) *Serratia marcescens* (shl).	This agent lowers AHL levels, which, in turn, leads to significant suppression of LasA protease, LasB elastase, caseinase, pyocyanin, pyoverdin, and the formation of biofilms.	Algburi et al. (2016)
	*Bacillus licheniformis* DAHB1,	*Vibrio parahaemolyticus*	It prevents biofilm growth in lab settings and decreases bacterial colonization and death in shrimp intestines.	Vinoj et al. (2014)
*Bifidobacterium*	*Bacillus licheniformis* T-1	*Aeromonas hydrophila*	The *ytnP* gene, which helps disrupt bacterial communication (quorum quenching), carries the instructions for building an acyl-homoserine lactone metallo-*β*-lactamase enzyme.	Kim et al. (2012)
	*Bacillus longum* ATCC15707	*Escherichia coli* 0157: H7	Inhibits AI-2 and reduces biofilm formation	Chen et al. (2019)
	*Lactobacillus acidophilus* 30SC	*Escherichia coli* O157: H7	Inhibits AI-2	Reis-Teixeira et al. (2017)
	*Lactiplantibacillus plantarum* M.2*, Latilactobacillus curvatus* B.67	*Listeria monocytogenes*	This agent blocks bacterial movement (swimming motility) and stops biofilms from forming, while also decreasing the expression of key genes linked to biofilm growth.	Reis-Teixeira et al. (2017)
	*Lactiplantibacillus plantarum* SBR04MA	Microbiota of activated sludge	Inhibits N-Hexanoyl-L-homoserine lactone (6-HSL)	Kim et al. (2018)
	*Lactiplantibacillus plantarum*	*Staphylococcus aureus*	Reduces expression of some genes involved in biofilm formation	Kampouris et al. (2018)
	*Lactobacillus acidophilus* GP1B	*Clostridium difficile*	Reduces production of AI-2 molecules	Yun et al. (2014)
	*Lactobacillus acidophilus* La-5	*Escherichia coli* 0157: H7	It disrupts quorum sensing (QS) molecules, which in turn reduces how well bacteria stick and form colonies.	Medellin-Peña and Griffiths (2009)
*Lactobacillus*	*Lactobacillus acidophilus* NCFM		It does not affect harmful bacteria; instead, it boosts the adherence of probiotics to gut cells by increasing *AI-2* in the *LuxS* system.	Medellin-Peña and Griffiths (2009)
	*Levilactobacillus brevis* 3 M004	*Pseudomonas aeruginosa*	Inhibits biofilm formation	Buck et al. (2009)
	*Lacticaseibacillus casei.*	*Streptococcus mutans*	Inhibits QS genes *vicKR* and *comCD*	Liang et al. (2022)
	*Lacticaseibacillus casei.* ATCC 393, *Limosilactobacillus reuteri* ATCC23272, *Lactiplantibacillus plantarum* ATCC14917 *Ligilactobacillus salivarius* ATCC11741	*Streptococcus mutans*	Inhibits acyl-homoserine lactone activity and blocks their synthesis	Wasfi et al. (2018)
	*Limosilactobacillus fermentum* Lim2	*Clostridioides difficile*	Reduces the *AI-2* in QS gene *luxS*	Yong et al. (2019)
	*Lactiplantibacillus plantarum* PA 100	*Pseudomonas aeruginosa*	Inhibits acyl-homoserine lactone activity and blocks their synthesis	Valdéz et al. (2005)
*Streptococcus*	*Streptococcus salivarius*	*Streptococcus mutans*	Inhibits biofilm formation in vitro when cultured with *Streptococcus mutans*	Tamura et al. (2009)
	*Streptococcus salivarius* K12	*Candida albicans*	It prevents *Candida albicans* from clumping together, forming biofilms, and changing its shape.	Mokhtar et al. (2021)
	*Streptococcus salivarius* 24SMB and *Streptococcus oralis* 89a	*Staphylococcus aureus, Staphylococcus epidermidis, Streptococcus pyogenes, Streptococcus pneumoniae, Moraxella catarrhaliss and Propionibacterium acnes*	This substance stops harmful bacteria in the upper respiratory tract from creating biofilms.	Bidossi et al. (2018)

### 3.6 Immunomodulatory effects

**Figure 3:**
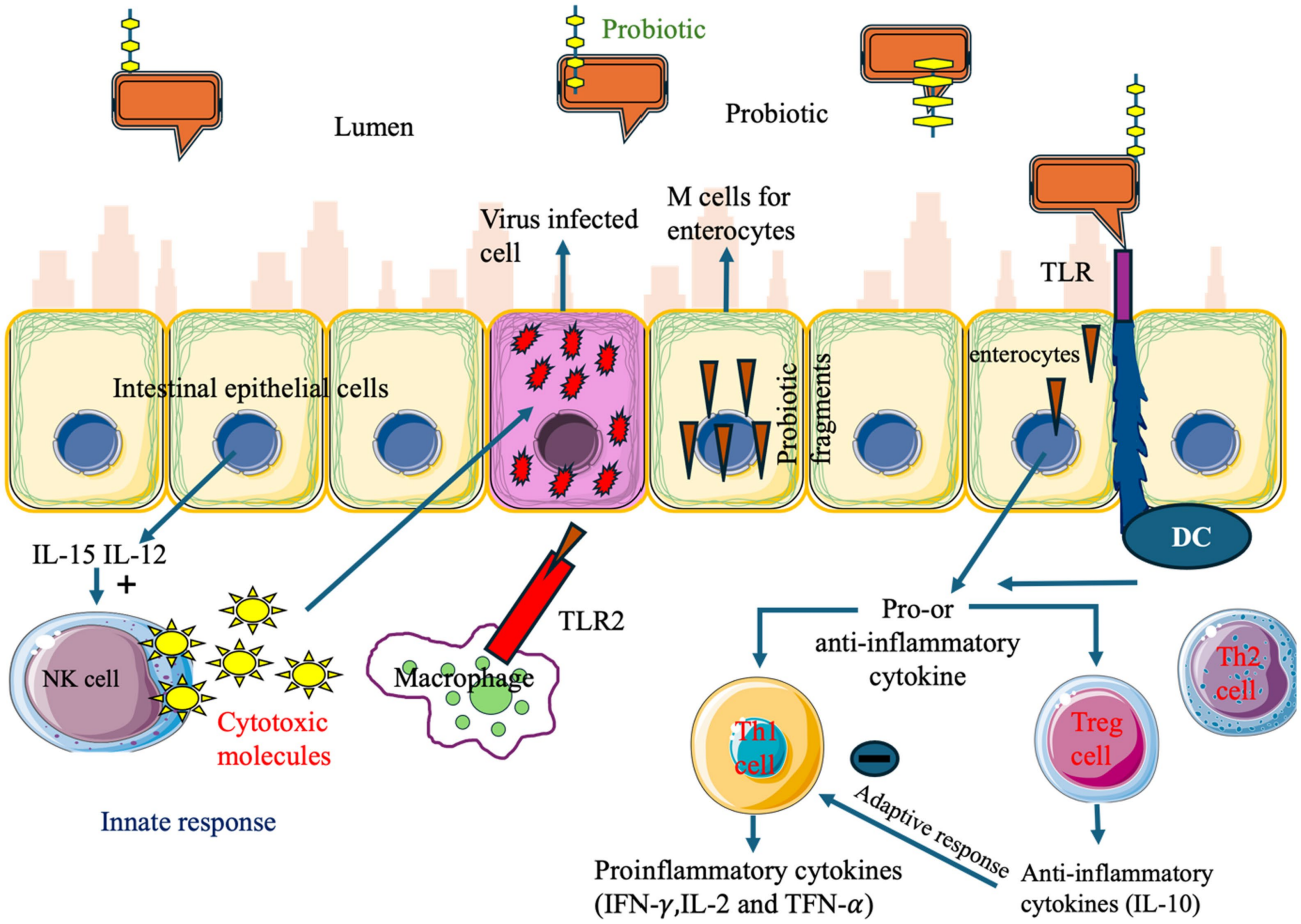
Mechanisms of probiotic-mediated modulation of intestinal barrier function, pathogen inhibition, and host immune responses.

Probiotics play a vital role in regulating both innate and adaptive immune responses, thereby strengthening host defence and reducing inflammation. In terms of mucosal immunity, probiotics enhance the secretion of secretory immunoglobulin A (sIgA), which neutralizes pathogens and prevents their adhesion to epithelial cells (Mazziotta et al., 2023; Gou et al., 2022; Joseph, 2022; Zhang J. et al., 2021). They also help maintain gut barrier integrity by reinforcing epithelial tight junctions and stimulating mucus production (Gieryńska et al., 2022; Shu et al., 2023; Zhou et al., 2024). Through cytokine regulation, probiotics modulate both pro- and anti-inflammatory cytokines to promote immune balance by reducing levels of IL-6 and TNF-*α* while increasing IL-10 to minimize allergic and chronic inflammatory responses (Figure 3) (Najafi et al., 2023; Zheng et al., 2025; Azad et al., 2018; Zhu et al., 2024; Abbaszadeh et al., 2024; Virk et al., 2024). Furthermore, probiotics prime innate immunity by activating macrophages, neutrophils, and dendritic cells, thereby enhancing the body’s ability to clear pathogens (Azad et al., 2018; Zhu et al., 2024). These immunomodulatory mechanisms highlight the therapeutic potential of probiotics in preventing infections, allergies, and chronic inflammatory diseases. Refer to Table 5 for the immunomodulatory effects of probiotics demonstrated in *in vivo* and *ex vivo* models.

**Table 5:** *In*/*ex vivo* immunomodulation effects of probiotics.

Studied model	Probiotics	Effects on Immunity	Reference
Older adults (over 75 years)	*Bifidobacterium longum* Bar33 and *Lactobacillus helveticus* Bar13	This intervention increases naive T cells, activated memory T cells, regulatory T cells, B cells, and natural killer (NK) cell activity, while simultaneously decreasing overall memory T cells.	Finamore et al. (2019)
Children	*Lacticaseibacillus paracasei* SD1	Decrease of *Streptococcus mutans* pathogens increase of salivary IgA	Pahumunto et al. (2019)
Piglets	*Limosilactobacillus reuteri* D8	Increase of goblet cells and antimicrobial peptides (AMPs), expressions of *Muc*2, *Lyz1*, and porcine *β-defensins* 1 (*pBD1*) Increase of CD3 + T cells, combined with increased expression of *IL-4* and *IFN-γ*	Wang et al. (2020)
Mice	*Limosilactobacillus fermentum* UCO-979C	It boosts intestinal IFN-γ, activates intestinal and peritoneal macrophages, and increases CD4 + T cells in Peyer’s patches. It also raises intestinal IL-6, intestinal IgA, and the number of mature B cells.	Garcia-Castillo et al. (2019)
Freshwater crayfish	*Lactobacillus acidophilus* and *Lactiplantibacillus plantarum*	Upregulation of cytokine gene families (*IL1β, IL8, IL10, and IL17F*), *proPO*, and *cytMnSOD*	Foysal et al. (2020)
Broilers challenged with *Escherichia coli*	*Lactobacillus acidophilus*	This intervention reduces the mortality rate caused by *Escherichia coli* infection. It also decreases levels of inflammatory markers like C-reactive protein, diamine oxidase, and endotoxin lipopolysaccharide at both 14 and 21 days. It improves gut barrier integrity by increasing the expression of tight junction proteins like Occludin and ZO-1 in the jejunum and ileum. Furthermore, it reduces inflammation by downregulating the expression of *iNOS, IL-8*, and *IL-1* beta in the jejunum of *Escherichia coli*-challenged birds at 21 days.	Wu et al. (2021)

## 4 Evidence from preclinical and clinical studies

The health-promoting potential of probiotics has been extensively evaluated across *in vitro* models, animal studies, and human clinical trials. These investigations provide strong evidence of their antimicrobial, immunomodulatory, and therapeutic properties.

### 4.1 *In vitro* studies

It has been proven by *in vitro* experiments that probiotics prevent pathogenic bacteria by means of competitive inhibition, antimicrobial component secretion, and changes in environmental conditions such as reducing pH that prevent growth (Ebrahiminejad et al., 2024; Silva et al., 2024). A number of pathogenic bacteria, in addition to *Escherichia coli,* are strongly suppressed by probiotics, consisting of *Clostridioides difficile*, *Salmonella typhimurium,* and *Enterococcus faecalis* (Prajapati et al., 2024; Raheem et al., 2021; Anjum et al., 2022). It is common for multispecies probiotic formulations to outperform single species in terms of *Escherichia coli* and *Klebsiella pneumoniae* inhibition, often to a significant extent (Huang et al., 2021; Kwoji et al., 2021; Ma et al., 2023). Although there may be cases specific to species in which interspecies competition may take place between probiotic strains, multispecies formulations such as poly-specific and biodiverse complexes are considered to have a wider range of action against pathogens (Mohammed et al., 2024; Zhao et al., 2023; Fijan et al., 2022; Knipe et al., 2021). *In vitro* studies have also demonstrated that a number of microorganisms involved in the development of dental caries, including *Streptococcus mutans*, are strongly suppressed by probiotics, suggesting a broader range of beneficial effects for oral health (Nair et al., 2024; Squarzanti et al., 2024; Di Stefano et al., 2023; Mokoena et al., 2021; Agbemavor and Buys, 2023).

### 4.2 Animal models

Animal studies provide insights into the systemic effects of probiotics in conditions such as metabolic syndrome, autoimmunity, and neurobehavioral disorders. Applications across livestock and aquaculture are summarized in Table 6.

**Table 6:** Animal applications of antimicrobial probiotics.

Animals	Probiotics	Form of administration	Effects	References
Poultry				
Broilers	*Lacticaseibacillus casei, Lactobacillus acidophilus,* and *Bifidobacterium*	Supplementing 1% of probiotics in water	It enhances growth, carcass quality, immune response, gut bacteria, and antioxidant levels.	Kaushik et al. (2024)
Laying hens	*Bifidobacterium* spp. and *Lacticaseibacillus casei.*	Feeding	The result is enhanced growth performance, increased egg weight, and improved feed efficiency.	Zhang L. et al. (2021)
Newly hatched chicks	*Lactiplantibacillus plantarum* LTC-113	Oral vaccination	It prevents *Salmonella* from colonizing by adjusting the expression of tight junction genes and inflammatory responses.	Lokapirnasari et al. (2019)
Chickens	*Lacticaseibacillus paracasei*	Feeding	Improving growth performance	Wang L. et al. (2018)
Broiler	*Lactobacillus johnsonii* BS15	Feeding	Preventing subclinical necrotic enteritis	Fesseha et al. (2021)
Chickens	*Bacillus licheniformis*	Feeding	It alleviates SNE-related gut injury, adjusts the intestinal microflora and barrier integrity, and regulates local immune responses in the gut.	Fesseha et al. (2021)
	*Clostridium butyricum*	Feeding	It promotes anti-inflammatory gene expression and tight junction protein genes, while inhibiting pro-inflammatory genes in *Clostridium perfringens* challenged chickens.	Wang H. et al. (2018)
Swine				
Weaning piglets	*Bacillus subtilis*, *Enterococcus faecium*	Liquid feed	Improve growth performance	Kan et al. (2021)
Piglets	*Lactiplantibacillus plantarum* (strains 22F and 25F) and *Pediococcus acidilactici* (strain 72 N)	Feeding	It mitigates the severity of ETEC challenges in weaned piglets	Huang et al. (2019)
Cattle				
Cattle	*Lactobacillus gallinarum* JCM 2011(T), *Streptococcus infantarius* subsp*. coli* HDP90246 (T), *Streptococcus salivarius* subsp. *thermophilus* ATCC 19258(T), and *Streptococcus equinus* ATCC 9812(T), *Saccharomyces cerevisiae*	Feeding	It enhances the development and blood chemistry of growing cattle.	Zhang et al. (2020)1
Dairy cows	*Saccharomyces cerevisiae, Bacillus subtilis, Bacillus licheniformis, Enterococcus faecium, Lactobacillus acidophilus,* and *Lactiplantibacillus plantarum,*	Feeding	It enhances reproductive performance, and increases both milk yield and the percentage of milk fat and protein.	Pupa et al. (2022)
	*Lacticaseibacillus rhamnosus, Pediococcus acidilactici*, and *Limosilactobacillus reuteri*	*Ex vivo* bovine endometrial explants	Reducing acute inflammation under *Escherichia coli* infection, decreasing *IL-8, IL-1β,* and *IL-6*	Yasmin et al. (2021)
Sheep				
Sheep, Lamb	*Enzimsporin*™ (*Bacillus subtilis* B-2998D, B-3057D, and *Bacillus licheniformis* B-2999D)	Feeding	Increasing body weight gain and improving intestinal microbiota	Merati and Towhidi (2022)
Fish				
Nile Tilapia (*Oreochromis niloticus*)	*Saccharomyces cerevisiae*	Feeding	Increasing growth performance and feed utilization indices	Genís et al. (2017)
Nile Tilapia (*Oreochromis niloticus*)	*Lactobacillus acidophilus* and *Enterococcus faecium*)	Feeding	It defends against *Aeromonas hydrophila* infection without hindering growth.	Devyatkin et al. (2021)
Common carp (*Cyprinus carpio*)	*Pediococcus pentosaceus*	Feeding	It enhances growth, boosts digestive enzyme activity, and strengthens blood-immune responses.	Islam and Rohani (2021)
Rohu fingerlings (*Labeo rohita*)	*Bacillus amyloliquefaciens* BN06, *Bacillus subtilis* WN07, and *Bacillus megaterium*	Feeding	It boosts growth and blood-immune health.	Cavalcante et al. (2020)
Shrimp				
Whiteleg shrimp, (*Litopenaeus vannamei*)	*Bacillus subtilis*, *Pediococcus pentosaceus,* and *Lactococcus lactis*	Feeding	It enhances growth, immunity, tissue health (histology), gene expression, digestive enzyme activity, and disease resistance.	Ahmadifar et al. (2020)
Pacific white shrimp (*Litopenaeus vannamei*)	*Bacillus subtilis* AQAHBS001	Feeding	It enhances growth, boosts immune response, and increases resistance to *Vibrio parahaemolyticus*.	Saravanan et al. (2021)

Overall, decisive results from new research detail probiotics complex physiological functions and applications. In the case of metabolic syndrome, probiotic treatment has managed to enhance fasting plasma glucose, triglycerides, cholesterol concentrations and, at the same time, improve gut barrier function (Won et al., 2020; Kewcharoen and Srisapoome, 2019). Moreover, in animal models this effect is associated with increased SCFA synthesis, the suppression of inflammation and, the reduction of markers of systemic inflammation element (Tain & Hsu, 2024; Zeighamy Alamdary et al., 2022; Breton et al., 2022; Zhang Z. et al., 2022). Concerning autoimmune diseases, especially in the EAE mice model, probiotics can decrease disease severity through immunotherapy (Tang et al., 2021). Also, in the Central Nervous System (CNS), gut *Lactobacillus helveticus*, an animal model of anxiety-like behavior reduction, is essential in gut-brain communications (Tu et al., 2023; Di Stefano et al., 2023; Džidić Krivić et al., 2025). In addition, within animal industries, probiotics also generate Grape Pomace Pectin (GPP), Fermented Products (FE), immunomodulation, and anti-infection properties across poultry, cattle, swine, fish, and shrimp, promoting growth performance and preventing disease (Kaushik et al., 2024; Saravanan et al., 2021). It is clear that probiotics can simultaneously support human body applications and biotechnological/industrial forces.

### 4.3 Human clinical trials

Recent human clinical trials have provided robust evidence supporting the therapeutic potential of probiotics across a range of health conditions. Randomized controlled trials (RCTs) have demonstrated that probiotic supplementation can significantly improve gastrointestinal function and modulate gut microbiota composition in human subjects, contributing to enhanced intestinal health (Zhang et al., 2025). Similarly, double-blind, placebo-controlled clinical trials have reported improvements in gastrointestinal symptoms and overall digestive well-being following probiotic intervention (Qu et al., 2025).

Regarding metabolic disorders, research conducted in a clinical setting suggests that probiotics may play a role in improving metabolic parameters, such as lowering fasting plasma glucose, serum triglycerides, and total cholesterol. The underlying mechanisms include altering gut microbiota composition, enhancing short-chain fatty acid production, and reducing systemic inflammation.

Probiotics have also demonstrated efficacy in the prevention and management of infectious diseases. Clinical evidence suggests that probiotic supplementation can reduce the incidence and severity of gastrointestinal infections, including antibiotic-associated diarrhea and *Clostridioides difficile* infection. Furthermore, meta-analyses of randomized controlled trials have highlighted the role of probiotics in reducing infection rates and improving clinical outcomes in hospitalized patients (Wu et al., 2023; Lee, Z., et al., 2023).

Further, human clinical studies have confirmed the ability of probiotics to boost the immunity of their host and thus decrease the likelihood of respiratory tract infection. Probiotics also help in maintaining vaginal microbiota homeostasis and lowering the chances of contracting urogenital infections through increasing colonization resistance to pathogens in females.

However, even with all these promising results, variations in the strains of the probiotics used, dosage, length of treatment, and the methodology of the studies conducted continue to pose major problems. Consequently, more extensive and well-conducted clinical studies need to be conducted to formulate standard guidelines.

Additionally, registered clinical trial databases such as ClinicalTrials.gov provide valuable insights into ongoing and completed probiotic intervention studies, highlighting the growing interest in their clinical applications.

## 5 Therapeutic applications in antimicrobial therapy

Initially considered as contaminants or ineffective commensals, emerging studies have demonstrated the promising therapeutic application of probiotics and as adjuncts or alternatives to conventional antimicrobial treatment or in vaccination. Thus, their spectrum of activity encounters gastrointestinal, urogenital, respiratory, and oral infections. All that in addition to preventing nosocomial infections and weakening antimicrobial resistance.

### 5.1 Gastrointestinal infections

Probiotics have been extensively studied for their therapeutic potential in gastrointestinal (GI) disorders, with particular interest in conditions associated with infections by *Clostridium difficile, Salmonella,* and pathogenic *Escherichia coli*. Treatment with probiotics, namely *Lactobacillus* species in the case of *Clostridium difficile* and *Saccharomyces boulardii*, significantly reduces the rate of relapse by returning the intestinal microbiota to its proper state and reinforcing the normal functions of the intestinal barrier (Al-shattrawi, 2024; Frost et al., 2023; Mori et al., 2023). In fact, up to a 50 percent decrease in the risk of the disease recurring was mentioned in meta-analyses (Frost et al., 2023; Mori et al., 2023). In the case of salmonellosis, the use of multi-strain probiotics causes a child’s illness to be less severe and persistent by reducing the duration and amount of pathogen-related diarrhea through competitive exclusion and efficient predatorily metabolites (Wombwell et al., 2021; Pal et al., 2023). In the context of *Campylobacter coli* infection, probiotics help maintain intestinal barrier integrity and promote IgA secretion, thereby reducing infection severity. Probiotics may also alleviate clinical symptoms associated with *Escherichia coli* diarrhea (Mehmood et al., 2023). This clinical scenario was demonstrated to be characterized by decreased frequency of diarrhea and a significant decrease in the patient’s level of suffering (Zhou et al., 2024, Guo et al., 2024; Zeise et al., 2021; Monika et al., 2021; Shu et al., 2023).

### 5.2 Urogenital infections

The supplementation of probiotics is useful in the treatment of other urogenital infections, especially recurrent UTIs and BV. Thus, the restoration of healthy vaginal flora and the prevention of the progression of the urogenital infection and further UTI recurrences attributed to *Lacticaseibacillus rhamnosus* GR-1 and *Limosilactobacillus fermentum* RC-14 strains have been nominated in several studies (Gupta et al., 2024; Reid et al., 2021; Qasemi et al., 2023; Pagar et al., 2024; Pessoa et al., 2022). The mechanism of the probiotics’ protection against the urogenital infection has been known to be based on the ability to suppress pathogens’ growth, secrete the antibacterial substances, block the entry of uropathogens to the epithelial cell walls, and boost the host immunity. In the case of BV, the received probiotic intervention has also improved the colporeal microbiocenosis by enhancing the number of *Lactobacillus* strains and maintaining the genuine balance within the vaginal microbiome (Mohan Kumar et al., 2022; MacPhee et al., 2010; Lehtoranta et al., 2022; Pendharkar et al., 2023). The application of probiotics will not raise the resistance of antibiotics, and the extended use in treating recurrent urogenital infections is justified.

### 5.3 Respiratory infections and asthma

Similarly, probiotics are a significant player of the gut–lung axis, affecting systemic immunity and respiratory health. Several meta-analyses have proven the ability of probiotic supplementation to diminish the incidence and duration of upper respiratory tract infections in both young and adult populations (Rashidi et al., 2021). For example, *Lacticaseibacillus rhamnosus* GG (LGG) has managed to decrease the probability of *rhinovirus* infections in adults (Liu, Y., et al., 2025). With asthma, the purpose is the alleviation of airway inflammation and improvement in lung function through the modulation of Th2-mediated immune reactions (Uwaezuoke et al., 2022). Currently, preliminary data available point to the amelioration of clinical outcomes in patients suffering from COVID-19, which also entails reduction in ICU admission rates (Synodinou et al., 2022).

### 5.4 Oral and dental infections

Thus, probiotics play a vital role in the maintenance of oral health, including the management of periodontal disease, dental caries, and post-surgical infections: (a) in periodontal disease, they help to minimize plaque accumulation and inflammation the application of probiotic strains like *Limosilactobacillus reuteri* contributed to the improvement of peri-implantitis outcomes (Inchingolo et al., 2023; Ram et al., 2024; Gheisary et al., 2022; Vazquez-Munoz and Dongari-Bagtzoglou, 2021); (b) in dental caries, they prevent *Streptococcus mutans* colonization and biofilm formation clinical trials showed that probiotic lozenges effectively reduce the incidence of caries (Luo et al., 2024; D’Agostino et al., 2024; Mickenautsch et al., 2023; D’Accolti et al., 2022), and (c) in post-surgical infections, probiotics aid in recovering the balance of the microbiota destroyed by antibiotics, thereby reducing the risk of postoperative complications (Matzaras et al., 2023; Yang et al., 2025).

### 5.5 Nosocomial (hospital-acquired) infections

Probiotics have also been explored as prophylactic agents in nosocomial infections, which are frequently multidrug-resistant and associated with substantial healthcare expenditures. For instance, LGG has significantly decreased the likelihood of nosocomial diarrhea in pediatric populations (Kalabalik-Hoganson et al., 2022). Therefore, synbiotics which are formulations combining probiotics and prebiotics and are superior to probiotics alone in reducing hospital-acquired infection, including pneumonia, bacteremia and bacteriuria, in critical, injured adult patients (Li C. et al., 2021; Chakraverty and Kundu, 2025). This is due in part to the greater ability of probiotics to both reestablish gut microbiota and to improve immunological competence, thereby increasing infection resistance (Dixit et al., 2021; Thananimit et al., 2022; Mazziotta et al., 2023). Despite the fact that the potency can depend on the type of probiotic, probiotics are widely recognized as safe, have no negative effects, and are perceived as a promising adjunct preventive strategy in hospitalized patients.

### 5.6 Probiotics as adjuncts in antibiotic therapy

Probiotics administered concurrently with antibiotics can decrease antibiotic-associated diarrhea and restore the gut microbiome more quickly. Meta-analyses indicate a 37–51% reduction in AAD incidence, with *Lacticaseibacillus rhamnosus* GG, *Saccharomyces boulardii,* and several *Bifidobacterium* species being especially effective (Kopacz and Phadtare, 2022; Mazhar et al., 2024). Probiotics were found to restore microbiome resilience by improving microbial diversity, impeding the proliferation of pathogens, and enhancing gut barrier functionality (Kopacz and Phadtare, 2022; Safarchi et al., 2025). Multi-strain and high-potency oral products in excess of 10 billion CFU (Colony Forming Units) daily were typically superior to single-strain or lower-potency pills (Mazhar et al., 2024). The use of probiotics is generally risk-free for the majority of individuals; however, there have been accounts of probiotic-induced bacteremia in immunocompromised individuals, indicating a need for increased monitoring in this population (Rossi et al., 2023).

### 5.7 Mitigation of antimicrobial resistance (AMR)

Apart from the aforementioned rarely harmful acquisitions, probiotics have been indicated to play a role in limiting the spread of antimicrobial resistance genes (ARG). Noteworthy, *Lactobacillus* and *Bifidobacterium* strains have demonstrated the ability to reduce the extent of ARG presence in the gut subsequent to antibiotic therapy (Duan et al., 2022). Clinical trials have shown evidence that probiotics can enhance the preservation of beneficial microbial populations, such as *Bacteroides*, and oppression of *Enterobacteriaceae*, which typically carry ARGs (Szajewska et al., 2024). An expected 71% lower incidence of antibiotic-associated *Clostridium difficile* infections was associated with probiotic supplementation, thus overall reducing the need for additional antibiotic consumption (Bagdadi et al., 2024; Bainum et al., 2023). These facts accentuate that probiotics affect not only the relief from antibiotic-related side effects but also participate in antimicrobial stewardship guarding against ARG dispersion.

## 6 Emerging technologies in probiotics

Recent advances in biotechnology have significantly expanded the scope of probiotics beyond traditional dietary supplementation. Novel approaches such as CRISPR-based genetic engineering, advanced encapsulation techniques, and targeted delivery systems are improving the stability, functionality, and therapeutic applications of probiotics.

### 6.1 CRISPR-based probiotic engineering

Moreover, CRISPR-Cas technology has revolutionized tools for microbial engineering and allows the precision editing of the genome of probiotic strains to enhance their functional potential (Patra, 2024; Nidhi et al., 2021). Moreover, engineered *Lactobacillus* strains with CRISPR-Cas systems can target solely antibiotic-resistant pathogens; that is, a targeted bactericidal effect inactivates resistance genes. The authors created CRISPR-modified *Escherichia coli* Nissle 1917 strains for targeting multidrug-resistant pathogenic strains as well (Muteeb et al., 2023; Agha et al., 2025). In another direction, using CRISPR elements for the synthetic biology of probiotic bacteria is shown, which allows them not only to sense the presence of the pathogen but also to generate a response signal in more (Arora, 2024; Patra, 2024). All of these perspectives, according to the authors, can transform probiotics from “livestock” of the human microbiota into a programmable therapeutic agent and help to design the basis of a very precise therapeutic approach in the future.

Thus, it is clear that the concept of “comprehensive” probiotics, including safety, metabolic activity, adhesion to the epithelium, and the production of active substances, require further study and development, and another study involving the genome-wide screening of already used strains according to the established criteria is underway.

### 6.2 Encapsulation techniques

The major drawback of probiotic therapy is ensuring the survival of microbial cells during storage and passage through the gastrointestinal tract. At the moment, there are many developed technologies based on the use of microencapsulation, which provides protection of probiotics from the damaging effects of various factors. While we are glad to see the creation of the GSM devices through the use of *Bacteroides* to produce mucin, Riedel’s team initially 3D printed the two mixed culture organisms and mucin and then the *Bacteroides* cells. Of the possible bioprinters that were used by Riedel’s group, the Envision TEC 3D-bioplotter can achieve precision in the micrometer range; however, as the materials are used, this estimate could change. Fluidized bed coating builds protective polymer layers around probiotic cells, increasing their viability during heat processing and storage. Emulsification embeds probiotics within lipid or polymer droplets, ensuring good protection and controlled release in the intestines (Song and Zhao, 2024). Providing a cost-effective and biocompatible method of safeguarding *Lactobacillus* strains, alginate-based systems, particularly calcium alginate beads (Lynch et al., 2022; Zhang and Cheng, 2022). Besides, biofilm-inspired encapsulation fixes probiotics in natural biofilm-like matrices that increase probiotic resistance and colonization (Li et al., 2023b). Thus, encapsulation both improves probiotic viability and allows for targeted functional metabolites delivery.

### 6.3 Targeted delivery mechanisms

Innovative delivery strategies are being investigated to further improve the therapeutic potential of probiotics by site-specifically releasing them within the gastrointestinal tract. pH-sensitive polymers that protect probiotics from gastric acidity and degrade in the small intestine are commonly used delivery systems (Pottie et al., 2024). Many types of nanocarriers, including liposomes, chitosan nanoparticles, and dendrimers, are capable of controlled site-specific delivery and can co-administer prebiotics or drugs for a synergistic effect (Costa et al., 2022). Also, through microencapsulation with bioactive compounds, namely prebiotics or plant-derived antimicrobials, both colonization efficiency and pathogen inhibition have been enhanced (Mocellin et al., 2003). Thus, enhanced probiotic viability and productivity from these types of designs and potential personalized probiotic therapy based on specific health demand.

## 7 Challenges and limitations

Despite strong evidence supporting probiotic benefits, several challenges limit their widespread therapeutic adoption. These challenges relate to strain-specificity, reproducibility, clinical standardization, safety concerns, and regulatory hurdles.

### 7.1 Strain-specific effects

The health benefits of probiotics are highly strain-specific; as a result, findings from one strain cannot automatically be applied to others within the same species (Ahmed et al., 2024; Jin et al., 2025). Certain strains of *Lacticaseibacillus rhamnosus*, particularly *Lacticaseibacillus rhamnosus* GG, have been shown to reduce the incidence of antibiotic-associated diarrhea (Szajewska and Kołodziej, 2015). Other factors influencing the therapeutic effectiveness of probiotics include dose, formula, and the host microbiota composition, further complicating reproducibility (Olatunji et al., 2024). Thus, careful strain identification and selection are essential in clinical applications.

### 7.2 Reproducibility and clinical translation (RCT)

The results of probiotics studies conducted *in vitro* and in animal models are not always consistent with human clinical findings. This inconsistency is mainly due to interspecies differences in the composition of the intestinal microbiota, diet, age, and body functioning (Talat and Khan, 2025). Although the combination of various strains in a multi-strain probiotic can be advantageous by expanding the area of therapeutic effectiveness, a loss of synergistic effects may also occur due to inter-strain competition (Soofi and Khan, 2024). To overcome this issue, it is necessary to conduct multicentre large RCT studies to correlate and confirm the effect of probiotics in patients.

### 7.3 Regulatory and manufacturing challenges

Regulatory mechanisms of probiotics remain incomplete at the international level because, given their food, rather than drug, status, probiotics are viewed as food, and regulatory gaps have ensued (Baindara et al., 2021). Sources of variation between research-based findings are numerous and include insufficient clinical substantiation for the label claims, as well as potential complications arising from probiotic viability loss during processing and storage and lower actual CFU counts relative to labelled amounts (Field et al., 2015). Henceforth, standardizing regulation and quality control guidelines is of critical importance for guaranteeing the credibility of the products.

### 7.4 Safety concerns

Probiotics are considered safe for use in healthy individuals; however, they might pose certain risks to immunocompromised or critically ill patients. Probiotic-related bacteremia and fungemia are uncommon but have been reported in isolated cases, particularly involving *Lactobacillus species* and *Saccharomyces boulardii* (De arauz et al., 2009; Heng et al., 2007; Cotter et al., 2005). Additionally some probiotic strains may carry antibiotic resistance genes, which raises a concern about the possibility of horizontal gene transfer within the gut microbiota. These facts underline the necessity for a comprehensive safety evaluation and genome sequencing of probiotic strains as a prerequisite for their clinical use.

### 7.5 Future research directions

In conclusion, overcoming the existing probiotic therapy challenges will be a complex journey. Strain-level genomics and proteomics are crucial for revealing functional genes and the uniqueness of strain effects. Synthetic biology can be used to engineer advanced and efficient targeted probiotics. Crosslinking probiotic intervention with patients’ unique host microbiome profile data could make custom probiotic interventions more efficient and practical. Scientists need to remain vigilant about potential long-term adverse effects and maintain systematic post-market monitoring, aiming them particularly closely at risk groups.
